# Identification and characterization of *NMNAT1* gene mutations in an Iranian patient with Leber congenital amaurosis 9

**DOI:** 10.1002/ccr3.9506

**Published:** 2024-10-22

**Authors:** Mostafa Neissi, Motahareh Sheikh‐Hosseini, Misagh Mohammadi‐Asl, Adnan Issa Al‐Badran, Mojdeh Roghani, Javad Mohammadi‐Asl, Kamele Jorfi

**Affiliations:** ^1^ Department of Genetics, Khuzestan Science and Research Branch Islamic Azad University Ahvaz Iran; ^2^ Department of Genetics, Ahvaz Branch Islamic Azad University Ahvaz Iran; ^3^ Noor‐Gene Genetic Laboratory Ahvaz Iran; ^4^ Pediatric Cell & Gene Therapy Research Center Tehran University of Medical Sciences Tehran Iran; ^5^ Department of Biology, College of Science University of Basrah Basrah Iraq; ^6^ Department of Medical Genetics, School of Medicine Ahvaz Jundishapur University of Medical Sciences Ahvaz Iran

**Keywords:** exome‐sequencing, Leber congenital amaurosis, mutation, *NMNAT1* gene

## Abstract

**Key Clinical Message:**

The discovery of compound heterozygous *NMNAT1* mutations (c.245T>C; p.Val82Ala and c.575A>G; p.Asp192Gly) provides a genetic explanation for Leber congenital amaurosis 9 in an Iranian patient. The proband's symptoms—including severe visual impairment, nystagmus, night blindness, and retinal degeneration—align with Leber congenital amaurosis 9 clinical features. This case underscores the value of exome‐sequencing in diagnosing rare genetic disorders and highlights its role in guiding personalized genetic counseling and potential treatments.

**Abstract:**

Leber congenital amaurosis is a severe early‐onset inherited retinal dystrophy. This study delves into the genetic basis of Leber congenital amaurosis, pinpointing compound heterozygous mutations in the *NMNAT1* gene as significant causative factors. While one mutation validates previous findings (c.245T>C; p.Val82Ala), the second (c.575A>G; p.Asp192Gly) proves novel, expanding the genetic landscape of Leber congenital amaurosis 9. Both mutations, inherited independently from nonconsanguineous parents, contribute to the intricate genetic basis of light on Leber congenital amaurosis 9 in this case. The identified mutations shed light on Leber congenital amaurosis genetics in the Iranian population, showcasing the efficacy of exome‐sequencing for molecular diagnoses in hereditary retinal degeneration. These findings provide valuable insights for tailored genetic counseling and potential therapeutic interventions.

## 1 INTRODUCTION

Inherited retinal dystrophies (IRDs) constitute a diverse group of disorders characterized by the progressive degeneration of photoreceptor cells within the retina, leading to varying clinical manifestations. Among these, Leber congenital amaurosis (LCA) emerges as a particularly severe form, showcasing profound visual impairment evident from birth. The hallmark of LCA is the early and drastic reduction or complete absence of electroretinogram (ERG) responses, typically noticeable within the initial year of life. This distinctive feature sets LCA apart as a highly impactful and challenging type of retinal degeneration.^1^, ^2^

On the contrary, retinitis pigmentosa (RP) represents a different facet of retinal dystrophy, initiating with the degeneration of rod photoreceptors followed by the subsequent demise of cone cells. RP manifests with symptoms such as night blindness, progressive deterioration of the visual field, and eventual central vision loss. Another variant, cone‐rod dystrophy (CRD), exhibits a unique pattern where cone cells bear the primary brunt of involvement, accompanied by concurrent or subsequent impairment of rod cells. Both LCA and CRD also share the occurrence of macular atrophy, commonly referred to as pseudocoloboma, though distinct from issues related to embryonic fissure closure.^3^, ^4^, ^5^

The intricate landscape of IRDs involves mutations in a staggering 281 genes, contributing to the pathogenesis of these disorders (RetNet, https://web.sph.uth.edu/RetNet/). A recent addition to this genetic mosaic is *NMNAT1*, a gene intricately involved in nicotinamide adenine dinucleotide (NAD) synthesis. Notably, *NMNAT1* has been identified as the causal factor in a specific subset of LCA cases, particularly those presenting with macular lesions.^6^, ^7^, ^8^, ^9^

*NMNAT1* is one of three nicotinamide mononucleotide adenylyltransferases responsible for converting nicotinamide mononucleotide and ATP into NAD^+^. NAD^+^ plays a crucial role in various redox reactions, particularly those significant in the central nervous system. While *NMNAT2* and *NMNAT3* are found in the Golgi apparatus and mitochondria, *NMNAT1* represents a nuclear isoform studied in the context of the Wallerian degeneration, slow (Wlds) mouse model. In this model, the fusion protein Wlds, comprising Ube4b and the entire coding sequence of Nmnat1, was found to exert a protective influence on axonal degeneration post‐neuronal injury, emphasizing the essential role of Nmnat1 activity in this phenomenon.^10^, ^11^, ^12^, ^13^, ^14^, ^15^

Research on *NMNAT1* has revealed intriguing findings, including the observation that homozygous null mutations in this gene, seen in both Drosophila melanogaster and murine models, lead to early lethality. However, heterozygous knockout mice exhibit normal development, suggesting a single functional copy of the gene suffices for typical developmental processes. Furthermore, a retinal knockout in Drosophila resulted in progressive retinal degeneration, indicating a potential link between NAD^+^ availability and axonal structure integrity. Interestingly, an inactive form of Nmnat1 was found effective in preventing photoreceptor degeneration, prompting the hypothesis that *NMNAT1* may serve an additional chaperone function beyond its recognized enzymatic role.^16^, ^17^

Exome‐sequencing, a valuable methodology, has played a pivotal role in identifying causative mutations in individuals with genetic disorders. This advanced technique allows targeted sequencing of the coding regions of genes, ensuring rapid, accurate, and cost‐effective identification of mutations responsible for single‐gene disorders.^18^, ^19^, ^20^ Given the compelling evidence at hand, we opted to utilize the exome‐sequencing technique to unravel the causative genetic defect in a nonconsanguineous Iranian family grappling with LCA. Through this approach, we successfully pinpointed previously undocumented compound heterozygous mutations residing within the *NMNAT1* gene. This discovery represents a significant stride forward in understanding the genetic basis of LCA9 in this particular familial context, shedding light on a novel mutation that contributes to the manifestation of the disorder.

## 2 MATERIALS AND METHODS

### 2.1 Subjects and clinical assessment

**FIGURE 1:**
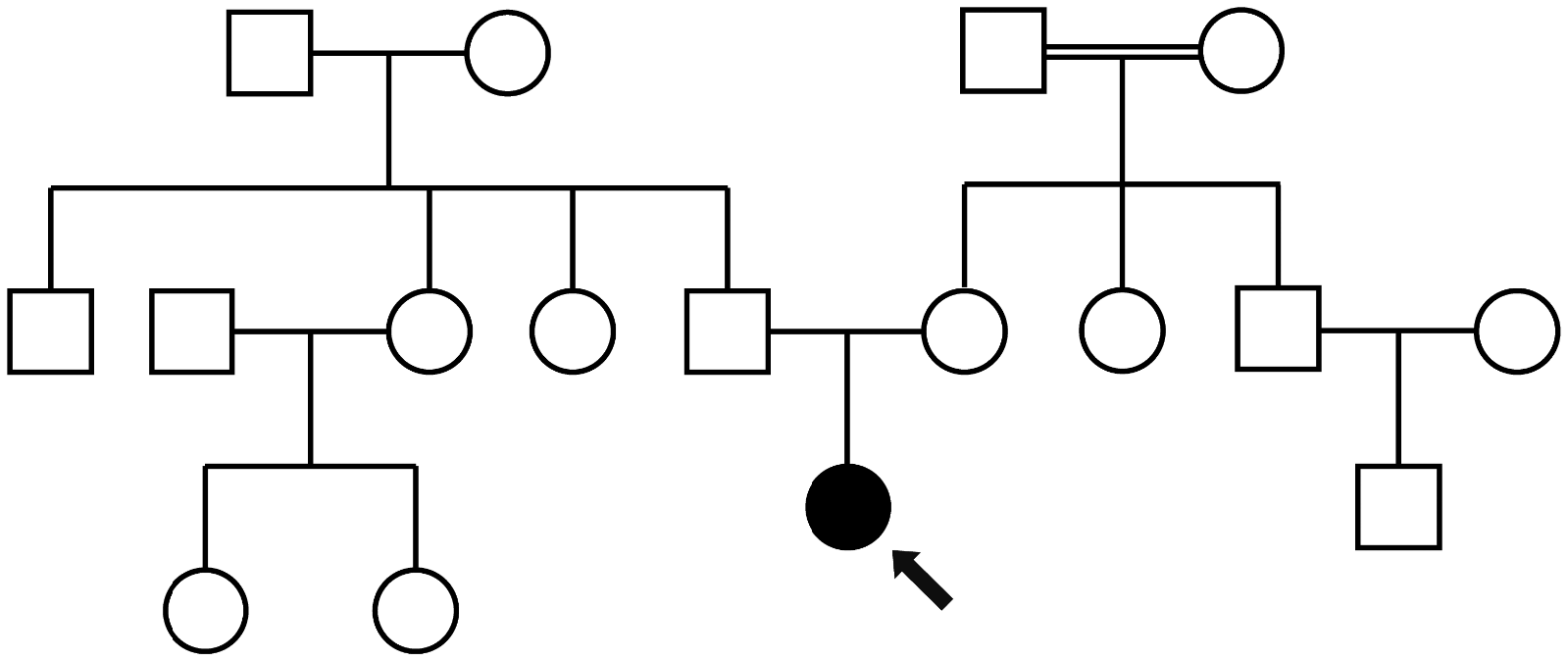
The pedigree of the studied family. Male members are denoted by squares, while females are represented by circles. Affected individuals are marked with enclosed symbols, and the proband is distinguished by an arrow.

The study focuses on a nonconsanguineous Iranian family grappling with LCA (Figure 1). The proband, a 6‐year‐old female patient, exhibited visual impairment and nystagmus from infancy. Clinical evaluation, including a thorough examination of ocular symptoms, electroretinography, and other relevant assessments, was initiated due to these symptoms, revealing severely impaired vision and confirming the suspicion of LCA.

### 2.2 DNA extraction

Peripheral blood samples, approximately 10 mL each, were obtained from the proband and accessible family members. Genomic DNA extraction was performed using the standard salting‐out protocol applied to blood leukocytes. The ensuing DNA samples underwent assessment for both quantity and quality through gel electrophoresis and nano‐drop analyses.

### 2.3 Exome‐sequencing

To pinpoint rare alleles linked to LCA, we employed exome‐sequencing for the enrolled patient. Library preparation was conducted using the SureSelect Human All Exon V6 kit, followed by sequencing on the Illumina HiSeq 2000 genome analyzer platform (Macrogen Inc., Seoul, Korea).

### 2.4 PCR and sanger sequencing

The design of PCR primers involved the utilization of OLIGO 7 (The sequences of the primers will be supplied upon request). Subsequent to primer design, PCR was executed utilizing 100 ng of genomic DNA, 12.5 μL of Master Mix from Amplicon Co., and 10 pmol of each primer, resulting in a total PCR volume of 25 μL. The amplification proceeded through an initial denaturation at 95°C for 5 min, followed by 35 cycles of denaturation at 95°C for 30 s, annealing at 60°C for 30 s, and extension at 72°C for 30 s, culminating in a final extension at 72°C for 5 min. The outcomes of Sanger sequencing were aligned with the reference genomic sequence and subjected to analysis using ChromasPro 2.1.3. Additionally, confirmation of the identified mutation was obtained through parental analysis and bidirectional Sanger sequencing. The purified DNA fragments underwent direct sequencing employing the Big Dye Terminator Cycle Sequencing Ready Reaction Kit on an Applied Biosystems 3500 DNA Analyzer. The resulting sequences were analyzed using Chromas and DNA Baser v4 software.

### 2.5 *In‐silico* pathogenicity assessing of genetic variants

To enhance the precision of our analysis, synonymous variants were excluded from consideration. Further refinement involved filtering based on allele frequency, utilizing data from the 1000 Genomes Project, Exome Sequencing Project (ESP), and Kaviar databases, coupled with the elimination of common variants (i.e., those with a minor allele frequency >0.01). Following this initial filtering, variants associated with diseases were identified and reported. Subsequently, the selected variants underwent comprehensive evaluation using multiple *in‐silico* prediction programs. This multistep approach enhances the robustness of pathogenicity assessment, ensuring a thorough and reliable analysis of genetic variants.

## 3 RESULTS

### 3.1 Clinical findings

The comprehensive clinical examination of the proband unearthed a myriad of debilitating manifestations. The foremost and perhaps most poignant among these was the revelation of severely impaired vision. The proband exhibited a profound reduction in visual acuity, consistent with the hallmark symptomatology of LCA. Additionally, the presence of nystagmus—a rapid, involuntary eye movement—further accentuated the complexity of the visual impairment experienced by the individual.

Further ophthalmological assessments, including fundus examination and ERG, revealed significant retinal degeneration and reduced or absent rod and cone responses, respectively. The proband also presented with night blindness and photophobia, common features associated with retinal dystrophies.

In addition to visual symptoms, the proband experienced significant difficulties with balance and coordination, often associated with advanced stages of retinal dystrophies. This was confirmed through vestibular testing, which demonstrated abnormal vestibular function. The proband also reported progressive loss of peripheral vision, which was evident in visual field testing, indicating a classic tunnel vision pattern.

A detailed medical history revealed that the proband had early‐onset visual disturbances, beginning in infancy, and had progressively worsened over time. The patient had no history of other systemic diseases or neurological issues.

Furthermore, systemic evaluations were conducted to rule out other possible conditions. Cardiological assessments, including echocardiography and electrocardiogram (ECG), revealed no abnormalities, ruling out any cardiac involvement. Laboratory tests, including a complete blood count, liver function tests, and kidney function tests, were all within normal limits. Neurological examination was unremarkable, with no signs of motor or cognitive impairment, further supporting the diagnosis of a primarily sensory disorder.

### 3.2 Genetic findings

Exome‐sequencing identified compound heterozygous mutations (GenBank: LC822758.1) within the *NMNAT1* gene (NM_022787.4) of the patient, which is notably associated with LCA9, as evidenced by the observed symptoms and the nature of the identified mutation. One mutation (c.245T>C; p.Val82Ala) had been previously reported,^21^ adding validation to the diagnostic journey, while the other mutation (c.575A>G; p.Asp192Gly) was novel, introducing a unique genetic signature to this familial narrative. The first mutation, identified as c.245T>C, involves a nucleotide substitution where thymine is replaced by cytosine at position 245 in exon 3. This change results in the substitution of the amino acid valine with alanine at position 82, denoted as p.Val82Ala. Concurrently, the second mutation, denoted as c.575A>G, signifies a nucleotide substitution where adenine is substituted by guanine at position 575 in exon 5, leading to the amino acid alteration p.Asp192Gly.

**FIGURE 2:**
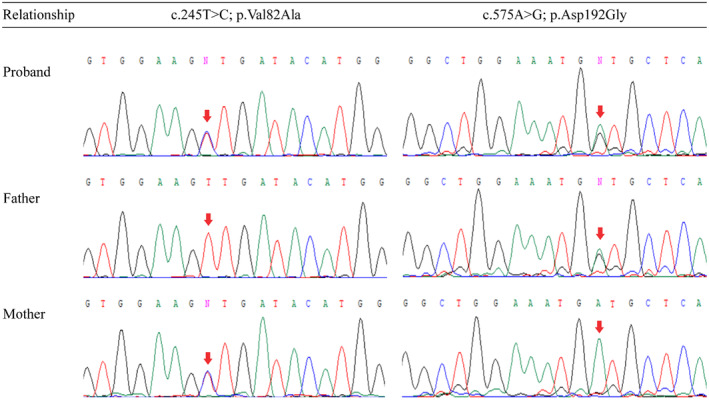
Results of Sanger sequencing are depicted, revealing compound heterozygous mutations in the proband. Specifically, the identified variants are c.245T>C; p.Val82Ala and c.575A > G; p.Asp192Gly of *NMNAT1*. The father exhibits the heterozygous c.575A>G; p.Asp192Gly mutation, while the mother carries the heterozygous c.245T>C; p.Val82Ala mutation.

Finally, the application of exome‐sequencing unveiled potential candidate variants within the gene, prompting a further layer of confirmation through direct Sanger sequencing. This meticulous validation process, conducted with an ABI3500 sequencer, solidified the identification of the c.575A>G; p.D192G mutation as inherited from the father and the c.245T>C; p.V82A mutation inherited from the mother (Figure 2). The absence of symptomatic manifestations in the carrier parents, despite the presence of the genetic anomaly, is consistent with the autosomal recessive inheritance pattern, where carriers do not manifest the disease. This highlights the importance of understanding inheritance patterns in genetic disorders.

### 3.3 *In‐Silico* analyses

Using both American College of Medical Genetics and Genomics (ACMG) guidelines and in‐silico predictor tools (Table 1), the identified mutations (*NMNAT1*: c.245T>C; p.Val82Ala and c.575A>G; p.Asp192Gly) were conclusively classified as likely pathogenic variants. Additionally, potential candidate variants within the gene were identified through exome‐sequencing and further confirmed by direct Sanger sequencing. The c.575A>G; p.D192G mutation was inherited from the father, and the c.245T>C; p.V82A mutation was inherited from the mother.

**TABLE 1:** Pathogenicity assessment of the variants identified by exome‐sequencing.

Gene	Variant	Polyphen‐2 HDIV score	CADD score	MetaLR score	MetaRNN score	FATHMM‐MKL score	MVP score	Mutation Taster
*NMNAT1*	V82A	1.000 (Probably damaging)	25.9 (Deleterious)	0.934 (Pathogenic moderate)	0.947 (Pathogenic strong)	0.9706 (Pathogenic supporting)	0.9903 (Pathogenic moderate)	Uncertain
*NMNAT1*	D192G	0.958 (Probably damaging)	25.2 (Deleterious)	0.8626 (Pathogenic strong)	0.6164 (Uncertain)	0.9618 (Uncertain)	0.9866 (Pathogenic moderate)	Benign supporting

The c.245T>C (p.Val82Ala) variant was classified as likely pathogenic based on several ACMG criteria. PP3 (strong evidence) was met, as the MetaRNN score of 0.947 is greater than the threshold of 0.939, indicating strong pathogenicity. PM5 (moderate evidence) was also satisfied because an alternative variant at the same position (chr1:9975720G>T; Val82Phe) is classified as likely pathogenic by ClinVar. Additionally, PM2 (supporting evidence) was confirmed as the variant was not found in gnomAD genomes, with good coverage (31.0), and the gnomAD exomes homozygous allele count of 0 is less than 2 for the autosomal recessive gene *NMNAT1* (coverage = 30.2). Furthermore, PP2 (supporting evidence) was applied since 66 out of 68 non‐VUS missense variants in *NMNAT1* are pathogenic (97.1%), exceeding the 80.8% threshold. Finally, PP5 (supporting evidence) was considered, as ClinVar classifies this variant as Likely Pathogenic, citing association with LCA9 and other conditions.

The c.575A>G (p.Asp192Gly) variant was also classified as likely pathogenic following ACMG criteria. PP3 (supporting evidence) was met since computational prediction tools unanimously supported a deleterious effect on the gene, with an aggregated score of 0.716. The score ranges for pathogenicity are: benign supporting (0–0.15), pathogenic supporting (0.7–0.8), pathogenic moderate (0.8–0.9), and pathogenic strong (0.9–1.0). PM2 (moderate evidence) was satisfied due to the extremely low frequency of the variant in gnomAD population databases, with no allele found in the gnomAD maximal non‐founder and founder subpopulations. PP2 (supporting evidence) was applicable because the missense variant is in a gene with a low rate of benign missense mutations and for which missense mutation is a common mechanism of disease. There are three times more pathogenic variants than benign variants for curated missense variants in this gene, with a high number of pathogenic missense variants (44) compared to benign ones.^5^

## 4 DISCUSSION

In the context of this study, we conducted exome‐sequencing with the aim of elucidating pathogenic mutations in a nonconsanguineous Iranian family exhibiting symptoms indicative of LCA. Through a systematic analysis of exome‐sequencing data, rigorous Sanger sequencing verification, and meticulous segregation analysis, we successfully identified compound heterozygous missense mutations in *NMNAT1* (NM_022787.4) in the affected daughter within the family. The specific mutations, namely c.245T>C; p.Val82Ala and c.575A>G; p.Asp192Gly, were found to be inherited from both parents, with one mutation transmitted from the father and the other from the mother. This multifaceted approach not only pinpointed the causative mutations but unequivocally confirmed LCA9 in the patient, providing a definitive diagnosis and elucidating the specific genetic basis underlying the observed clinical manifestations.

*NMNAT1*, situated on the genomic locus 1p36.22, is characterized by five exons. This gene encodes the nuclear nicotinamide mononucleotide adenylyltransferase 1, a key enzyme in NAD biosynthesis. Its principal role involves catalyzing the conversion of nicotinamide mononucleotide and ATP into NAD^+^, making it an essential player in cellular energy metabolism.^17^, ^22^, ^23^ NMNAT1 plays a pivotal role as a fundamental constituent of the Wlds fusion protein, responsible for orchestrating a pronounced delay in axonal degeneration. This delay in axonal degeneration holds significant implications for neurological diseases, as it has the potential to decelerate the disease progression and extend overall survival. Notably, the enzymatic activity of NMNAT1 is indispensable for the Wlds‐mediated axonal protection.^12^, ^24^, ^25^ Intriguingly, even when considered in isolation, devoid of all other Wlds sequences, NMNAT1 alone exhibits substantial efficacy in providing robust axonal protection, as evidenced in transgenic mouse models.^26^ In the model organism Drosophila melanogaster, the complete inactivation of the nmnat isoform results in lethality, accompanied by a profound degeneration of photoreceptors.^17^ In mice, the absence of both Nmnat1 alleles leads to embryonic lethality, and a heterozygous knockout manifests with substantially diminished enzyme activity levels.^16^ These observations strongly corroborate the overarching conclusion that NMNAT1 stands as an indispensable protein crucial for the sustenance of both function and survival within the realm of photoreceptor neurons.

*NMNAT1*, a gene situated in close proximity to the previously mapped LCA9 locus through rigorous linkage analysis within a consanguineous Pakistani family,^27^ emerged as a pivotal player in the etiology of LCA. This noteworthy revelation was substantiated by multiple research groups, each independently confirming *NMNAT1* as a causative gene for LCA. The collective findings of these distinct investigations were concurrently disseminated in the pages of Nature during the pivotal year of 2012,^6^, ^7^, ^8^, ^9^ underscoring the significance and veracity of NMNAT1's association with this debilitating ocular disorder. Several documented mutations within the *NMNAT1* gene have been noted for their impact on the enzymatic activity of the mutant protein. Specifically, a homozygous missense mutation (c.25G>A, p.Val9Met) was discerned to exhibit a diminished enzymatic activity in comparison to the wild‐type counterpart, as evidenced in fibroblast cells derived from the proband.^7^ Yet another instance of significance within the spectrum of *NMNAT1* mutations is represented by a homozygous missense mutation (c.769G>A, p.Glu257Lys), where a parallel outcome akin to the previously mentioned mutation was discerned, this time in red blood cells.^8^ Recently, an array of novel or previously identified mutations in *NMNAT1* has been documented through various research endeavors.^28^, ^29^, ^30^, ^31^ Notably, among the reported variants, the mutation denoted as c.769G>A; p.Glu257Lys has emerged as the most prevalent. Despite the extensive exploration of *NMNAT1* mutations, it is noteworthy to mention that the two mutations, namely c.769G>A; p.Glu257Lys and c.25G>A; p.Val9Met, have not been corroborated within the context of this specific family. This highlights the diverse landscape of *NMNAT1* mutations and underscores the importance of comprehensive genetic analyses to discern the unique genetic makeup of individual families affected by these variations.

The mutation identified as c.769G>A, which has been observed to impact the enzymatic activity of *NMNAT1*. In the course of our investigation, the compound heterozygous mutations (c.245T>C and c.575A>G) were forecasted to exhibit deleterious effects. Considering the heterogeneous nature of LCA as a group of inherited diseases, we hypothesize that the compound heterozygous mutations may potentially serve as a causative factor, influencing the activity of *NMNAT1*. Moreover, beyond its hypothetical involvement in the genesis of LCA, there is a compelling line of reasoning suggesting that this genetic alteration might extend its influence to impact the activity of *NMNAT1*, further accentuating its significance in the intricate molecular landscape associated with this ocular disorder. As we navigate the intricate pathways of genetic variations, our findings pose intriguing questions that underscore the need for continued exploration and deeper investigations into the precise mechanisms and implications of the compound heterozygous mutations within the multifaceted spectrum of LCA.

Identifying the mutation responsible for the development of LCA in patients is imperative for delivering precise genetic counseling and shaping the trajectory of gene‐specific therapies. Recent investigations into gene therapy, particularly centered around *RPE65*, have not only enriched our understanding but also provided a robust foundation for advancing the field of LCA gene therapy.^32^, ^33^, ^34^ Given the genetic diversity inherent in LCA, we utilized exome‐sequencing to explore causative mutations, aiming to achieve a precise molecular diagnosis. Our findings demonstrated the efficacy of this approach in efficiently narrowing down candidate genes and identifying genetic variants in small pedigrees associated with Mendelian inheritance diseases.

## 5 CONCLUSION

The compound heterozygous *NMNAT1* mutations comprising c.245T>C; p.Val82Ala and c.575A>G; p.Asp192Gly have been successfully identified as the causative mutations in a nonconsanguineous Iranian family affected by LCA9. This discovery significantly contributes to our understanding of the genetic basis of LCA within this specific population. Moreover, our study highlights the efficiency of employing exome‐sequencing as a robust method for conducting molecular diagnoses in cases of hereditary retinal degeneration. The comprehensive coverage provided by this sequencing technique proves instrumental in uncovering rare and complex mutations, underscoring its utility in advancing our ability to unravel the genetic underpinnings of inherited eye disorders. The identification of specific mutations, such as those in this study, not only aids in providing tailored genetic counseling but also holds promise for guiding the development of targeted therapeutic interventions in the realm of hereditary retinal degeneration.

## AUTHOR CONTRIBUTIONS

**Mostafa Neissi:** Conceptualization; investigation; writing – original draft. **Motahareh Sheikh‐Hosseini:** Investigation; writing – review and editing. **Misagh Mohammadi‐Asl:** Investigation; writing – original draft. **Adnan Issa Al‐Badran:** Investigation. **Mojdeh Roghani:** Investigation; writing – review and editing. **Javad Mohammadi‐Asl:** Investigation. **Kamele Jorfi:** Investigation; writing – review and editing.

## FUNDING INFORMATION

No external funding was received for this study.

## CONFLICT OF INTEREST STATEMENT

The authors declare no conflict of interest.

## CONSENT

Written informed consent was obtained from the patient to publish this report in accordance with the journal's patient consent policy.

## Data Availability

The data that support the findings of this study are available from the corresponding author upon reasonable request.

## References

[1] Berger W , Kloeckener‐Gruissem B , Neidhardt J . The molecular basis of human retinal and vitreoretinal diseases. Prog Retin Eye Res. 2010;29(5):335‐375. doi:10.1016/j.preteyeres.2010.03.004 20362068

[2] den Hollander AI , Black A , Bennett J , Cremers FP . Lighting a candle in the dark: advances in genetics and gene therapy of recessive retinal dystrophies. J Clin Invest. 2010;120(9):3042‐3053. doi:10.1172/JCI42258 20811160 PMC2929718

[3] Sahel J , Bonnel S , Mrejen S , Paques M . Retinitis pigmentosa and other dystrophies. Dev Ophthalmol. 2010;47:160‐167. doi:10.1159/000320079 20703049

[4] Hamel CP . Cone rod dystrophies. Orphanet J Rare Dis. 2007;2:7. doi:10.1186/1750-1172-2-7 17270046 PMC1808442

[5] Heckenlively JR , Foxman SG , Parelhoff ES . Retinal dystrophy and macular coloboma. Doc Ophthalmol. 1988;68(3‐4):257‐271. doi:10.1007/BF00156432 3042323

[6] Chiang PW , Wang J , Chen Y , et al. Exome sequencing identifies NMNAT1 mutations as a cause of Leber congenital amaurosis. Nat Genet. 2012;44(9):972‐974. doi:10.1038/ng.2370 22842231

[7] Falk MJ , Zhang Q , Nakamaru‐Ogiso E , et al. NMNAT1 mutations cause Leber congenital amaurosis. Nat Genet. 2012;44(9):1040‐1045. doi:10.1038/ng.2361 22842227 PMC3454532

[8] Koenekoop RK , Wang H , Majewski J , et al. Mutations in NMNAT1 cause Leber congenital amaurosis and identify a new disease pathway for retinal degeneration. Nat Genet. 2012;44(9):1035‐1039. doi:10.1038/ng.2356 22842230 PMC3657614

[9] Perrault I , Hanein S , Zanlonghi X , et al. Mutations in NMNAT1 cause Leber congenital amaurosis with early‐onset severe macular and optic atrophy. Nat Genet. 2012;44(9):975‐977. doi:10.1038/ng.2357 22842229

[10] Berger F , Lau C , Dahlmann M , Ziegler M . Subcellular compartmentation and differential catalytic properties of the three human nicotinamide mononucleotide adenylyltransferase isoforms. J Biol Chem. 2005;280(43):36334‐36341. doi:10.1074/jbc.M508660200 16118205

[11] Coleman MP , Freeman MR . Wallerian degeneration, wld(s), and nmnat. Annu Rev Neurosci. 2010;33:245‐267. doi:10.1146/annurev-neuro-060909-153248 20345246 PMC5223592

[12] Mack TG , Reiner M , Beirowski B , et al. Wallerian degeneration of injured axons and synapses is delayed by a Ube4b/Nmnat chimeric gene. Nat Neurosci. 2001;4(12):1199‐1206. doi:10.1038/nn770 11770485

[13] Avery MA , Sheehan AE , Kerr KS , Wang J , Freeman MR . Wld S requires Nmnat1 enzymatic activity and N16‐VCP interactions to suppress Wallerian degeneration. J Cell Biol. 2009;184(4):501‐513. doi:10.1083/jcb.200808042 19237597 PMC2654119

[14] Conforti L , Fang G , Beirowski B , et al. NAD(+) and axon degeneration revisited: Nmnat1 cannot substitute for Wld(S) to delay Wallerian degeneration. Cell Death Differ. 2007;14(1):116‐127. doi:10.1038/sj.cdd.4401944 16645633

[15] Yahata N , Yuasa S , Araki T . Nicotinamide mononucleotide adenylyltransferase expression in mitochondrial matrix delays Wallerian degeneration. J Neurosci. 2009;29(19):6276‐6284. doi:10.1523/JNEUROSCI.4304-08.2009 19439605 PMC6665489

[16] Conforti L , Janeckova L , Wagner D , et al. Reducing expression of NAD+ synthesizing enzyme NMNAT1 does not affect the rate of Wallerian degeneration. FEBS J. 2011;278(15):2666‐2679. doi:10.1111/j.1742-4658.2011.08193.x 21615689

[17] Zhai RG , Cao Y , Hiesinger PR , et al. Drosophila NMNAT maintains neural integrity independent of its NAD synthesis activity. PLoS Biol. 2006;4(12):e416. doi:10.1371/journal.pbio.0040416 17132048 PMC1665629

[18] Di Resta C , Galbiati S , Carrera P , Ferrari M . Next‐generation sequencing approach for the diagnosis of human diseases: open challenges and new opportunities. EJIFCC. 2018;29(1):4‐14. 29765282 PMC5949614

[19] Neissi M , Al‐Badran AI , Mohammadi‐Asl J . Exome sequencing identifies a novel GUCY2D mutation in an Iranian family with Leber congenital amaurosis‐1: a case report. Egypt J Med Hum Genet. 2022;23(1):9. doi:10.1186/s43042-022-00217-9

[20] Al‐Badran RA , Al‐Badran AI , Mabudi H , Neissi M , Mohammadi‐Asl J . Detection of an FYCO1 nonsense mutation in an affected patient with autosomal recessive cataract (CTRCT18): a case report. Egypt J Med Hum Genet. 2022;23(1):52. doi:10.1186/s43042-022-00272-2

[21] Bedoukian EC , Zhu X , Serrano LW , Scoles D , Aleman TS . NMNAT1‐associated cone‐rod dystrophy: evidence for a spectrum of foveal maldevelopment. Retin Cases Brief Rep. 2022;16(3):385‐392. doi:10.1097/ICB.0000000000000992 32150116

[22] Hughes KT , Ladika D , Roth JR , Olivera BM . An indispensable gene for NAD biosynthesis in salmonella typhimurium. J Bacteriol. 1983;155(1):213‐221. doi:10.1128/jb.155.1.213-221.1983 6305909 PMC217671

[23] Schweiger M , Hennig K , Lerner F , et al. Characterization of recombinant human nicotinamide mononucleotide adenylyl transferase (NMNAT), a nuclear enzyme essential for NAD synthesis. FEBS Lett. 2001;492(1–2):95‐100. doi:10.1016/s0014-5793(01)02180-9 11248244

[24] Gillingwater TH , Ribchester RR . Compartmental neurodegeneration and synaptic plasticity in the Wld(s) mutant mouse. J Physiol. 2001;534(Pt 3):627‐639. doi:10.1111/j.1469-7793.2001.00627.x 11483696 PMC2278742

[25] Araki T , Sasaki Y , Milbrandt J . Increased nuclear NAD biosynthesis and SIRT1 activation prevent axonal degeneration. Science. 2004;305(5686):1010‐1013. doi:10.1126/science.1098014 15310905

[26] Sasaki Y , Vohra BP , Baloh RH , Milbrandt J . Transgenic mice expressing the Nmnat1 protein manifest robust delay in axonal degeneration in vivo. J Neurosci. 2009;29(20):6526‐6534. doi:10.1523/JNEUROSCI.1429-09.2009 19458223 PMC2697066

[27] Keen TJ , Mohamed MD , McKibbin M , et al. Identification of a locus (LCA9) for Leber's congenital amaurosis on chromosome 1p36. Eur J Hum Genet. 2003;11(5):420‐423. doi:10.1038/sj.ejhg.5200981 12734549

[28] Corton M , Nishiguchi KM , Avila‐Fernández A , et al. Correction: exome sequencing of index patients with retinal dystrophies as a tool for molecular diagnosis. PLoS One. 2016;11(3):e0153121. doi:10.1371/journal.pone.0153121 27031522 PMC4816403

[29] Siemiatkowska AM , van den Born LI , van Genderen MM , et al. Novel compound heterozygous NMNAT1 variants associated with Leber congenital amaurosis. Mol Vis. 2014;20:753‐759. 24940029 PMC4043607

[30] Jin X , Qu LH , Meng XH , Xu HW , Yin ZQ . Detecting genetic variations in hereditary retinal dystrophies with next‐generation sequencing technology. Mol Vis. 2014;20:553‐560. 24791140 PMC4000715

[31] Coppieters F , Van Schil K , Bauwens M , et al. Identity‐by‐descent‐guided mutation analysis and exome sequencing in consanguineous families reveals unusual clinical and molecular findings in retinal dystrophy. Genet Med. 2014;16(9):671‐680. doi:10.1038/gim.2014.24 24625443

[32] Maguire AM , Simonelli F , Pierce EA , et al. Safety and efficacy of gene transfer for Leber's congenital amaurosis. N Engl J Med. 2008;358(21):2240‐2248. doi:10.1056/NEJMoa0802315 18441370 PMC2829748

[33] Bainbridge JW , Smith AJ , Barker SS , et al. Effect of gene therapy on visual function in Leber's congenital amaurosis. N Engl J Med. 2008;358(21):2231‐2239. doi:10.1056/NEJMoa0802268 18441371

[34] Cideciyan AV , Aleman TS , Boye SL , et al. Human gene therapy for RPE65 isomerase deficiency activates the retinoid cycle of vision but with slow rod kinetics. Proc Natl Acad Sci USA. 2008;105(39):15112‐15117. doi:10.1073/pnas.0807027105 18809924 PMC2567501

